# GSNOR is essential for nitric oxide homeostasis and involved in aflatoxin biosynthesis and pathogenicity in *Aspergillus flavus*

**DOI:** 10.1128/aem.01408-25

**Published:** 2025-12-16

**Authors:** Dongyue Chen, Yuan Jiao, Xinping Wang, Fang Tao

**Affiliations:** 1 School of Life Sciences, Anhui Agricultural University 12486, Hefei, China; 2 National Engineering Laboratory of Crop Stress Resistance Breeding, Anhui Agricultural University 12486, Hefei, China; Chalmers tekniska hogskola AB, Gothenburg, Sweden

**Keywords:** *Aspergillus flavus*, GSNOR, nitric oxide, aflatoxin, pathogenicity

## Abstract

Nitric oxide (NO) is an important biological signaling molecule. *S*-nitrosoglutathione reductase (GSNOR), a master regulator of NO signaling, regulates various biological processes. However, little is known about the role of GSNOR in *Aspergillus flavus*. Here, we identified a gene encoding GSNOR in this aflatoxigenic fungus and demonstrated that GSNOR shows activity during the critical life cycle stages, including spore germination, hyphal growth, and conidiogenesis. We found that GSNOR plays a crucial role in NO homeostasis, as GSNOR deletion resulted in significantly elevated NO levels and heightened sensitivity to exogenous NO stress. GSNOR also participated in multiple biological processes in *A. flavus*; for that, GSNOR deletion impaired conidia germination, reduced growth, decreased conidiogenesis and sclerotial development, attenuated virulence on kernels, and notably decreased aflatoxin production. Furthermore, we demonstrated that GSNOR is important for reactive oxygen species (ROS) balance, as its deletion significantly elevated mycelial ROS levels and made the strain more sensitive to oxidative stress.

**IMPORTANCE:**

*Aspergillus flavus* is a notorious saprophytic filamentous fungus, with its production of carcinogenic aflatoxins posing serious threats to food safety and human health. Aflatoxin contamination prevention and control have long been a global challenge. In previous studies, we observed that nitric oxide (NO) significantly inhibits the aflatoxin production by *A. flavus*. This study further investigated the role of the key regulatory enzyme *S*-nitrosoglutathione reductase (GSNOR) in the NO signaling pathway. Our findings indicate that GSNOR is crucial for maintaining both NO homeostasis and reactive oxygen species (ROS) balance and plays an essential role in fungal development, pathogenicity, and aflatoxin biosynthesis. These results highlight the potential of targeting components in the NO signaling pathway, such as GSNOR, as a novel strategy for the early prevention of aflatoxin contamination in food.

## INTRODUCTION

Nitric oxide (NO), as a cellular signaling molecule, regulates numerous physiological and differential processes. In mammals, it is crucial for cell growth, apoptosis, vasodilation, neurotransmission, and the immune response (1). In plants, it influences growth and development, as well as in responses to biotic and abiotic stresses (2, 3). NO has also been found to play a role in the regulation of fungal growth, differentiation, and secondary metabolism (4–6). For example, in *Neurospora crassa*, NO could inhibit conidiation and promote vegetative growth (7). In *Fusarium graminearum*, the NO produced upon infection of plant roots could modulate the expression of genes associated with fungal virulence and development (8). In *Aspergillus nidulans*, NO has been found to stimulate the production of sexual cleistothecia and decrease sterigmatocystin production (9). NO also plays an important role in fungal response to abiotic and biotic stresses (10, 11).

NO homeostasis is crucial for maintaining optimal vitality in organisms. At low levels, NO acts as a signaling molecule. However, excessive NO can react with various reactive oxygen species (ROS) to form reactive nitrogen species (RNS), including NO, nitrous oxide (N_2_O), and peroxynitrite (ONOO^−^), as well as other types of nitrogen-containing reactive free radicals (12). These highly reactive RNS can trigger nitrosative stress, disrupting cellular proteins, DNA, and lipids, ultimately causing cell toxicity. *S*-nitrosoglutathione reductase (GSNOR) plays a crucial role in NO homeostasis (4). RNS derived from NO can react with the major cellular antioxidant glutathione, forming *S*-nitrosoglutathione (GSNO). Through the catalytic action of GSNOR, GSNO is further broken down into GSSG and NH_3_, contributing to denitrification (13).

GSNOR is an important regulator in the NO signaling pathway for its key role in modulating the levels of GSNO and the bioavailability of NO. Especially, GSNO, a major cache of NO bioactivity, serves as a primary NO donor for protein *S*-nitrosation within the cell. The –NO group can reversibly covalently bind to the thiol group (Cys-SH) of cysteine residues in proteins, forming *S*-nitrosothiols. This modification typically alters protein activity or function and is crucial for the adaptive regulation of organisms (14, 15). By catalyzing the breakdown of GSNO, GSNOR regulates the *S*-nitrosylation of proteins, which affects cellular signaling and homeostasis. In mammals, GSNOR is critical for various physiological and pathological processes, including immune function, inflammation, development, and cancer progression (16–18). In plants, GSNOR has been shown to regulate growth, development, and responses to various biotic and abiotic stresses (15, 19, 20). In contrast, research on GSNOR in microorganisms is relatively limited. In filamentous fungi, GSNOR from *A. nidulans* was first characterized through recombinant expression (21). In *Cryptococcus neoformans*, GSNOR eliminates GSNO metabolic activity and attenuates virulence (22). In *Ganoderma lucidum*, GSNOR is involved in the regulation of the biosynthesis of the secondary metabolite ganoderic acid under heat stress (23).

*Aspergillus flavus* is notorious for producing aflatoxins, highly carcinogenic mycotoxins. In a previous study, we demonstrated that exogenous NO could affect aflatoxin production in *A. flavus*. Increased endogenous NO levels from *fhbA* deletion reduced aflatoxin production (24). However, the function of GSNOR, as an important regulator in the NO signaling pathway, remains unknown in *A. flavus*. In this study, we identified a GSNOR from *A. flavus* and investigated the effects of GSNOR deletion on endogenous NO levels. The mycelial growth, development, pathogenicity, aflatoxin production, and oxidative stress response of the mutant were then evaluated.

## MATERIALS AND METHODS

### Fungal strains and culture conditions

*A. flavus* NRRL 3357 was used as the wild-type strain in this work. Wild-type and derivative transformants were maintained and cultured on potato dextrose agar (PDA, 39 g/L, BD Difco, USA) or CDA (Czapek-Dox Broth [BD Difco, USA] + 1.5% agar) medium. Wickerham medium (25), with half the concentration of corn steep liquor, was used to observe the formation of sclerotia. Yeast extract and sucrose medium (YES) (6 g/L yeast extract, 150 g/L sucrose, 15 g/L agar) was used for aflatoxin production analysis.

*Saccharomyces cerevisiae* strain FY834 was used for recombination-based cloning. The strain was refreshed on YPD agar medium (yeast extract, 10 g/L; glucose, 20 g/L; peptone, 20 g/L). Yeast transformants were selected on Sc-U medium (yeast nitrogen base, 1.7 g/L; ammonium sulfate, 5 g/L; casein hydrolysate, 5 g/L; adenine hemisulfate salt, 20 mg/L; glucose, 20 g/L).

### Construction of GSNOR knockout, complementation, and localization strains

The gene deletion mutants were generated using a double-fluorescence knockout system as described (26) with minor modifications. The pUM-GFP vector serves as the backbone, with the orientation of the tef1-GFP sequence being opposite to our previous study. For the gene knockout construct, DNA fragments of the ~1.0 kb flanking regions of the target gene were amplified with primer pairs *gsnor*_KO_5f/gsnor_KO_5r and *gsnor*_KO_3f/*gsnor*_KO_3r. The phleomycin-resistant gene *Ble* and *RFP* gene fused cassette, *Ble-RFP*, was amplified from the pDHBG vector (27) with the primers Pbr-f/Pbr-r. The pUM-GFP vector was linearized by *Bam*H I. All fragments were mixed and transformed into the yeast strain FY834. The homologous recombination plasmid products were verified by sequencing and named pKO-*gsnor*. This plasmid was transformed into wild-type NRRL 3357 using the *Agrobacterium tumefaciens*-mediated transformation (ATMT) method (28). All phleomycin-resistant transformants were first screened by fluorescence. The transformants emitting only red fluorescence were then selected for homogeneous nuclei (HMN) strains using negative screening double PCR with primers P*gsnor*-f/P*gsnor*-r and Ptub-f/Ptub-r.

The complemented strain was constructed using a site-specific integration system (29). Simply, a 2.65-kb DNA fragment containing a 0.66-kb upstream sequence, a full-length *gsnor* gene coding region, and a 0.57-kb downstream sequence was amplified from NRRL 3357 genomic DNA using the primers Com-*gsnor*-f/Com-*gsnor*-r, and then cloned into the T-DNA region of pUM vector (29) using a yeast recombination cloning approach to generate pUM::Com*-gsnor* vector. The T-DNA region also included a 1.03-kb *sdh2* left flanking fragment (containing the *sdh2* mutation site H249L) amplified with P101/P104, and a 1.07-kb *sdh2* right flanking fragment amplified with P105/P106, which were used for homologous recombination after transformation to insert the exogenous fragment into the *sdh2* locus. The pUM::Com*-gsnor* vector was reintroduced to the conidia of the *gsnor* deletion mutant through ATMT. The transformants were screened on CDA plates with 300 μg/mL carboxin and validated by double PCR with primer pair P100-F/Ps-*gsnor*-R and Ptub-f/Ptub-r. The positive transformant was named Com-*gsnor*.

The subcellular localization strain was also constructed using the site-specific integration system. The process is similar to that of complementation strain construction, except that GSNOR was fused with GFP for expression. The constructed plasmid pUM::Sub::*gsnor* was then transformed into the wild type. The transformants were first screened by green fluorescence and then validated by double PCR with primer pair P100-F/Ps-*gsnor*-R and Ptub-f/Ptub-r. The positive transformant was named Sub::*gsnor*.

Primers used in this section are listed in Table S1 at https://doi.org/10.6084/m9.figshare.30770495.

### Phenotypic characterization

To assess the fungal growth, 10 μL of conidial suspension (1 × 10^6^ spores/mL) was inoculated onto fresh CDA and PDA media, followed by incubation at 30°C for 5 days. The colony images were photographed at 7 days post-inoculation (dpi). To evaluate sporulation, conidia of the tested strains were harvested from CDA and PDA agar plates at 7 dpi with 0.01% Triton X-100 and counted with a hemocytometer.

Conidial germination assay was conducted as follows: coverslips were placed in a Petri dish (90 mm). Then, 5 mL YES medium was poured into the dish. One microliter of conidial suspension (1 × 10^6^ spores/mL) was inoculated onto each slip and cultured at 37°C. Conidial germination was observed by microscopic examination of at least 1,000 conidia per replicate at each time point. For sclerotia analysis, 1 × 10^2^ spores were centrally seeded onto the Wickerham medium (WKM) plates and incubated in the dark at 30°C for 10 days. The conidia were then washed off the plates with 75% alcohol, and the remaining sclerotia were counted under a microscope. These experiments were repeated three times with three replicates each, and representative results from one experiment are shown.

### Pathogenicity assay

A laboratory kernel infection assay was performed as previously described (26). Undamaged maize kernels were sterilized with 75% ethanol and 1% NaClO for 5 min in turn. The kernels were immersed in a conidial suspension (2 × 10^6^/mL) and shaken at 70 r/min at 30°C for 30 min. The kernels were then incubated at 30°C for 10 days. The kernels immersed in distilled water served as the mock, and three replications were conducted for each test. Infection was designated as visible mycelia and conidia on the surface of the kernel. The rate of infection was calculated by dividing the infected area by the kernel surface area (26). Spores were also harvested and counted with a hemacytometer.

### NO and ROS detection assay

Intracellular NO was visualized using DAF-FM DA (Beyotime, Shanghai, China) as follows. Coverslips were gently inserted at an angle into CDA medium, with spores inoculated at the junction between the coverslip and the medium. After incubation at 30°C for 14 and 16 h, the mycelia grown on the coverslips were immersed in 5 µM DAF-FM DA solution, followed by incubation at 37°C for 30 min in the dark. The mycelia were then washed three times with PBS solution (pH 7.4). Fluorescence images were captured using a laser confocal microscopy, with an excitation wavelength of 495 nm and an emission wavelength of 515 nm. Fluorescence intensity was analyzed using the ImageJ software.

Intracellular ROS was visualized using DCFH-DA (Beyotime, Shanghai, China) staining. Mycelial culture and staining methods were the same as those used in the NO assay, except that during laser confocal microscopy observation, the excitation wavelength was 488 nm and the emission wavelength was 525 nm.

### Enzyme activity assays

GSNOR activity was measured by monitoring the decomposition of NADH as described (30) with minor modifications. Briefly, 1 × 10^4^ spores were inoculated on the YES plate and cultured at 28°C for 2 days. Mycelia were harvested, ground in liquid nitrogen, and dispersed in 1 mL of 20 mM Tris-HCl (pH 8.0). After thorough mixing, the sample was centrifuged at 12,000 rpm for 5 min at 4°C, and the supernatant was collected for further analyses. In a 96-well plate, 300 μL of assay mix contained 15 µL of the test sample, 20 mM of Tris-HCl (pH 8.0), 200 μM of NADH, and 400 μM of GSNO. The reaction was incubated at 25°C, and the absorbance at 340 nm was monitored for 10 min after the addition of NADH using an EnSpire microplate reader (PerkinElmer, Waltham, USA). The decomposition rate was corrected for background NADH oxidation of each extract in the absence of GSNO. Protein concentration was determined using the Bradford protein assay kit (Beyotime, Shanghai, China). The enzyme activities of catalase (CAT), peroxidase (POD), and superoxide dismutase (SOD) were determined using the relevant kits (Nanjing Jiancheng Bioengineering Institute, Nanjing, China) according to the manufacturer’s instructions. The kit catalog numbers were A007, A084, and A001, respectively.

### Aflatoxin analysis

Aflatoxin extraction from mycelia was carried out according to our previously described method (24). Each tested strain, containing 1 × 10⁴ spores, was inoculated on sterile cellophane sheets overlaid on the YES plate and cultured at 28°C for 4 days. The fungal biomass was scraped from the plates and weighed, then extracted by incubation with 5 mL of methanol/water (7:3) at room temperature, with shaking at 200 rpm for 2 h. The supernatant was then collected by centrifugation at 3,000 × *g* for 10 min at room temperature and filtered through a syringe filter (0.22 µm, Alltech, Nicholasville, KY, USA). Aflatoxin extraction from infected maize kernels followed the methodology outlined in our previous studies (31).

The quantitative analysis of AFB1 was carried out using UPLC-MS/MS, as previously described (24). Briefly, separation of the AFB1 was carried out on an ACQUITY UPLC BEH C 18 (2.1 mm × 50 mm, 1.7 µm, Waters, MA, USA). The elution solutions used were (A) 0.1% formic acid in water and (B) 0.1% formic acid in MeOH. The solutions were pumped at a flow rate of 0.3 mL/min, and A/B = 60:40 was applied. Waters MassLynx V4.2 SCN986 analysis software (Waters, MA, USA) was used to control the LC/MS/MS system and to acquire and process data. The mass spectrometer was operated in the multiple reaction monitoring mode. The main MS parameters were optimized and finally set as follows: capillary voltage, 3.5 kV; cone voltages, 72 V; desolvation temperature, 500°C; desolvation gas flow rate, 1,000 L/h; cone gas, 150 L/h; precursor ion (m/z), 313; and quantification ion (m/z), 285.

### RNA isolation and quantitative PCR

Conidial suspension (1 × 10^4^ spores) was seeded on sterile cellophane sheets overlaid on the YES plate and incubated at 28°C for 3 days in the darkness. The mycelia of *A. flavus* were collected for total RNA isolation using Trizol reagent (TaKaRa, Dalian, China) according to the manufacturer’s instructions. For quantitative analysis of *gsnor* gene expression at different developmental stages, conidia were harvested using 0.01% Triton X-100, followed by centrifugation at 10,000 rpm to collect the spores. The samples cultured in liquid YES medium at 28°C for 10 and 24 h were designated as germinating hyphae and mature hyphae, respectively.

One microgram of total RNA was reverse transcribed into cDNA using a HiScript III RT SuperMix for qPCR (+gDNA wiper) (Vazyme, Co. Ltd., China), with the reaction incubated at 42°C for 2 min, followed by a PCR program of 37°C for 15 min, 85°C for 5 s. qPCR was conducted using the ChamQ Universal SYBR qPCR Master Mix (Vazyme, Co. Ltd., China), in a final volume of 20 μL, consisting of 10 μL 2× ChamQ Universal SYBR qPCR Master Mix, 0.4 μL of each primer (10 mM), and 2 μL cDNA. The qPCR program included an initial denaturation at 95°C for 30 s, followed by a 2-step PCR, 40 cycles of 95°C for 10 s and 60°C for 30 s.

The expression of *gsnor* was assessed using primer pairs q-gsnor-f/q-gsnor-r. The expression of *aflR* and *aflS* was quantified using primer pairs q-aflR-f/q-aflR-r and q-aflS-f/q-aflS-r, respectively. The *β-tubulin* gene, amplified with primer pairs q-tub-f/q-tub-r, served as the endogenous control. Five biological replicates were assessed for each sample. The relative levels of expression were calculated using the comparative CT (2^−ΔΔCT^) method. Primers used in this section are listed in Table S1 at https://doi.org/10.6084/m9.figshare.30770495.

### Statistical analysis

All experimental results were reported as mean ± standard deviation (SD). Statistical analyses were performed with GraphPad Prism 8.0 software (GraphPad Software, San Diego, CA, USA) using one-way ANOVA. The significance level was set at *P* < 0.05.

## RESULTS

### Characterization of GSNOR in *A. flavus*

**Fig 1:**
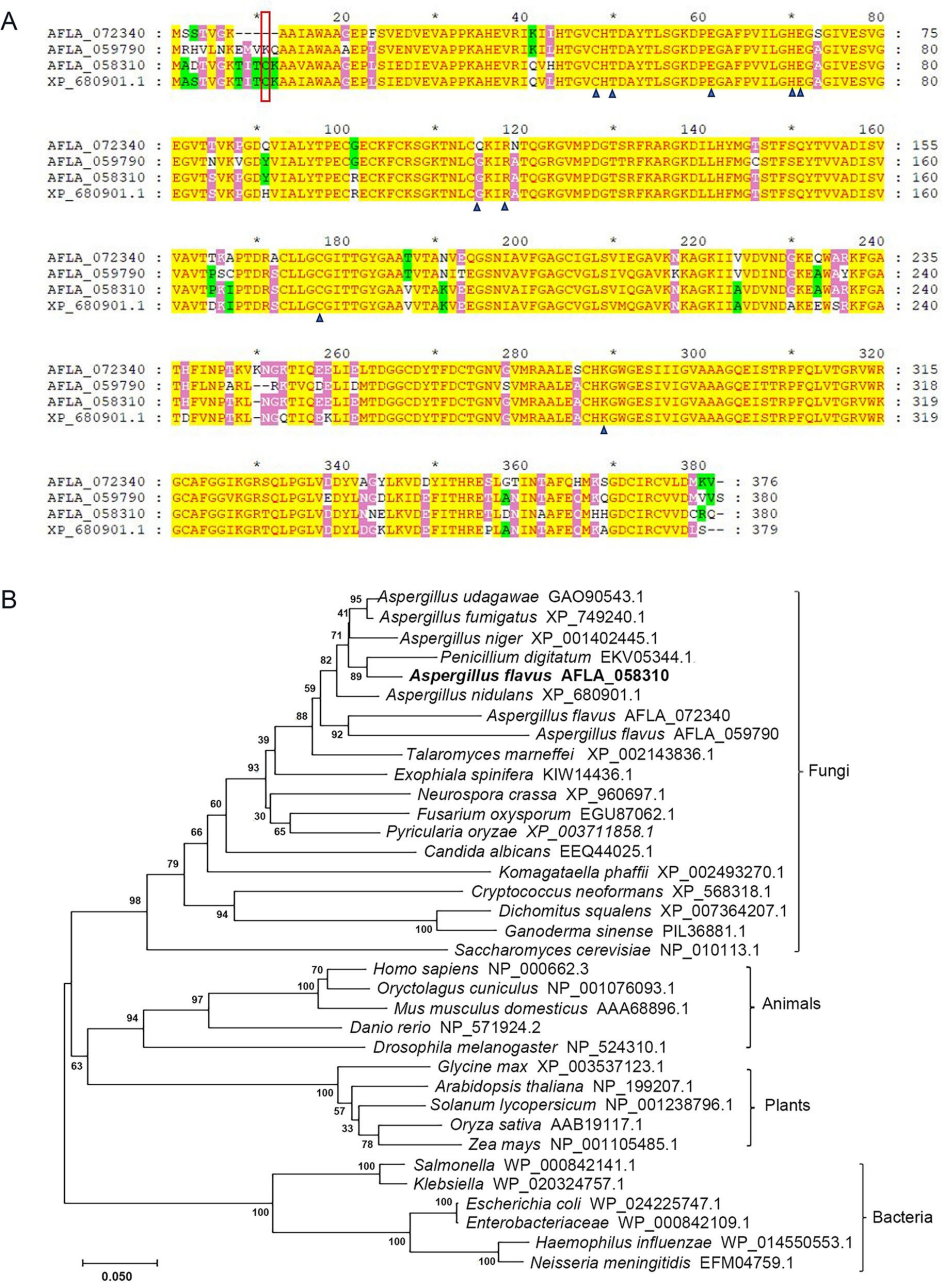
Analysis of GSNOR amino acid sequences. (**A**) Amino acid sequence analysis of GSNOR homologs in *A. flavus* and *A. nidulans*. The closed (▲) black arrowheads designate the highly conserved amino acid residues. The red box designates the conserved Cys11. (**B**) Alignment and phylogenetic analysis of the amino acid sequences of GSNOR homologs in *A. flavus* and other eukaryotic and prokaryotic species.

Homologous searches were performed in the *A. flavus* NRRL 3357 genome database (https://www.jcvi.org/) using the known GSNOR amino acid sequence of *A. nidulans* (XP_680901.1). Three orthologous proteins encoded by AFLA_058310, AFLA_059790, and AFLA_072340 were identified in *A. flavus*, with sequence similarities to *A. nidulans* GSNOR (21) of 92.9%, 81.8%, and 84.3%, respectively (Fig. 1A). GSNOR, initially identified as glutathione-dependent formaldehyde dehydrogenase (GS-FDH [13] or FALDH [32]), is a widespread enzyme also known as a class III alcohol dehydrogenase. Here, the amino acid sequences of GSNOR, ADH3, GS-FDH, or FALDH proteins in animals, plants, bacteria, and fungi, including the three orthologs in *A. flavus,* were aligned (see Fig. S1 at https://doi.org/10.6084/m9.figshare.30768476). Phylogenetic analysis revealed that the three orthologs in *A. flavu*s clustered within the fungal group (Fig. 1B).

In *Homo sapiens* (33, 34), *Arabidopsis thaliana* (35), and some fungi (21, 36), several residues coordinated to the active site zinc, the binding of substrates, and the ligand of the binary coenzyme complex are highly conserved. Correspondingly, some of the conserved amino acid residues, including Cys48, Thr50, Glu61, His70, Glu71, Arg118, Cys177, and Lys283, are all found in the three orthologous proteins encoded by AFLA_058310, AFLA_059790, and AFLA_072340, respectively. And AFLA_072340 contains Gln115, similar to Gln111 in *Homo sapiens*. However, in the closely related species *A. nidulans*, Cys11 has been confirmed to play an important role in regulating GSNOR activity by site-directed mutagenesis (21). Among the three homologs in *A. flavus*, only the one encoded by AFLA_058310 contains Cys11 (Fig. 1A), suggesting that it may be a GSNOR protein. The predicted *gsnor* gene (AFLA_058310) is 1,422 bp in length, consisting of five introns and six exons, and encodes a 380-amino acid protein.

### GSNOR exhibits GSNO reductase activity and is expressed at all developmental stages

The *gsnor* deletion mutant was generated using *Agrobacterium*-mediated homologous recombination (26). The principle of constructing the gene knockout strain is shown in Fig. S2A at https://doi.org/10.6084/m9.figshare.30768476. The entire coding region of *gsnor* was replaced with *Ble-RFP* fusion cassette. The transformant that exhibited red fluorescence was a putative null mutant (see Fig. S2B at https://doi.org/10.6084/m9.figshare.30768476). To exclude the mutant with heterogeneous nuclei, the null mutant with HMN was identified through negative PCR, no band for *gsnor* gene and only one band for *β-tubulin* gene could be amplified in this mutant (see Fig. S2C at https://doi.org/10.6084/m9.figshare.30768476).

To further confirm that the defects observed in Δ*gsnor* were ascribed to the deletion of *gsnor*, a complemented strain Com-*gsnor* was generated by introducing a genomic copy of *gsnor* into Δ*gsnor* using a site-specific integration system (see Fig. S2D at https://doi.org/10.6084/m9.figshare.30768476). Carboxin-resistant transformants were verified by positive PCR. The results showed that one band of approximately 1.9 kb was amplified, respectively, from the complemented strain but not from Δ*gsnor* (see Fig. S2E at https://doi.org/10.6084/m9.figshare.30768476), indicating that *gsnor* had been inserted into the specific site.

**Fig 2:**
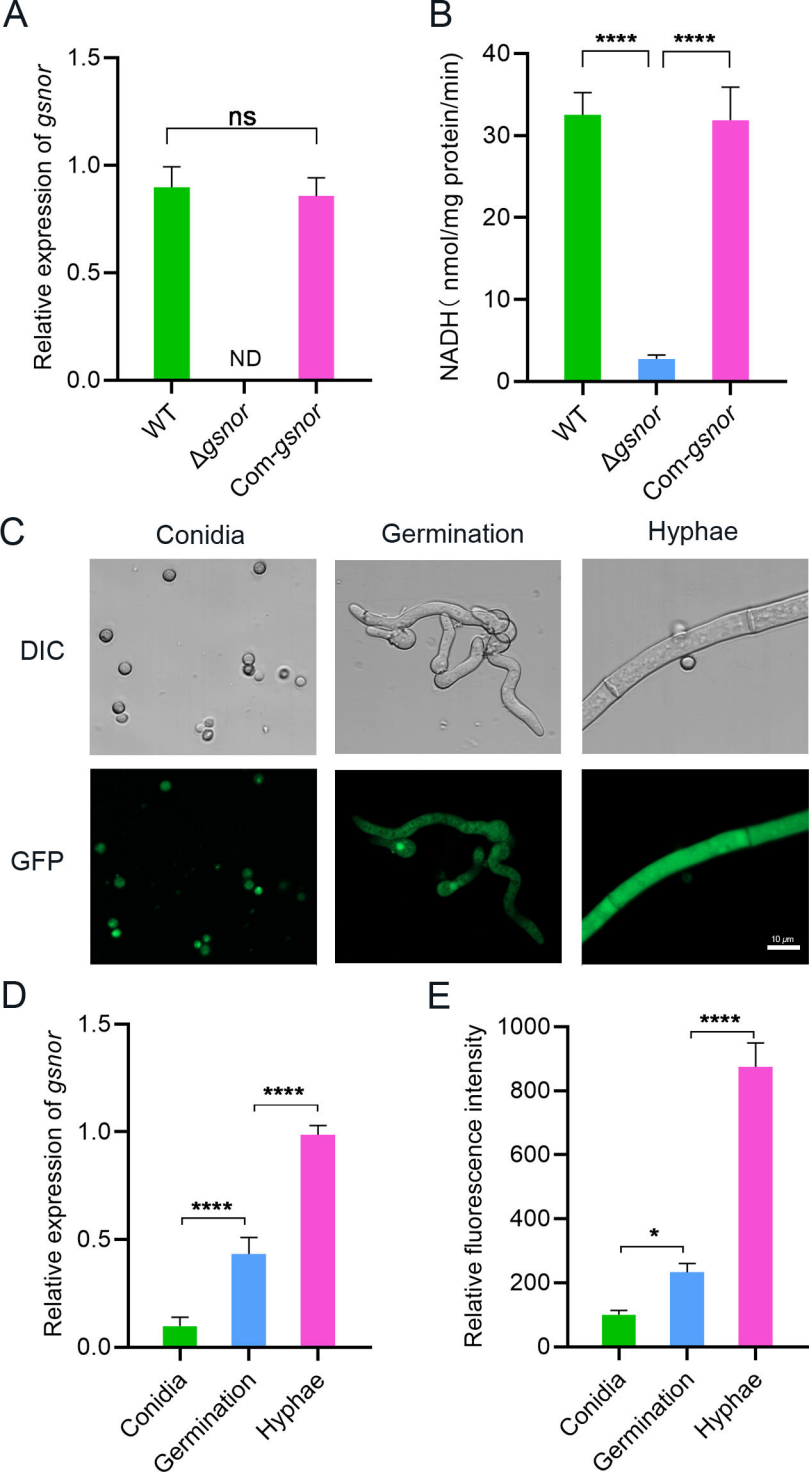
GSNOR mutation and subcellular localization. (**A**) Influence of *gsnor* deletion on the expression of *gsnor*. (**B**) Influence of *gsnor* deletion on GSNOR enzyme activity. (**C**) Expression of GSNOR-GFP in *A. flavus*. The conidia, germinated conidia, and hyphae of the Sub::*gsnor* strain were observed using a laser scanning confocal microscope. (**D**) Relative fluorescence intensity of GFP. (**E**) Quantitative analysis of *gsnor* gene expression in different developmental stages. Asterisks indicate significant differences, *P < 0.05, ****P < 0.0001. “ND” stands for “not detected.” “ns” indicates not significant.

Further RT-qPCR demonstrated that *gsnor* was expressed in both wild type and Com-*gsnor* without a significant difference, but was undetected in the Δ*gsnor* mutant (Fig. 2A). Moreover, GSNO reductase activities and *gsnor* transcription levels were consistent across the three genotypes (Fig. 2B). In the Δ*gsnor* strain, only minimal background GSNOR enzyme activity was observed, which could be attributed to the residual endogenous NADH in the mycelia. These results not only confirm the successful knockout of the *gsnor* gene in the mutant strain but also validate that the AFLA_058310 gene cloned in this study encodes GSNOR.

Here, the expression of GSNOR in *A. flavus* was also analyzed using a subcellular localization strain. A fusion expression vector containing *gsnor* with its own promoter and the GFP gene was constructed and transformed into the *A. flavus* wild-type strain (see Fig. S2F and G at https://doi.org/10.6084/m9.figshare.30768476). The expression of GSNOR in the Sub::*gsnor* strain at different development stages was observed (Fig. 2C). The results indicated that GSNOR was mainly localized to the cytoplasm and was expressed during the conidia, spore germination, and hyphal growth stages. Quantitative analysis revealed the highest expression levels of *gsnor* in the hyphae (Fig. 2D), followed by germination, with the lowest levels observed in the conidia. This was further corroborated by GFP fluorescence analysis, which confirmed high GSNOR expression in the hyphae (Fig. 2E).

### GSNOR is required for NO homeostasis

**Fig 3:**
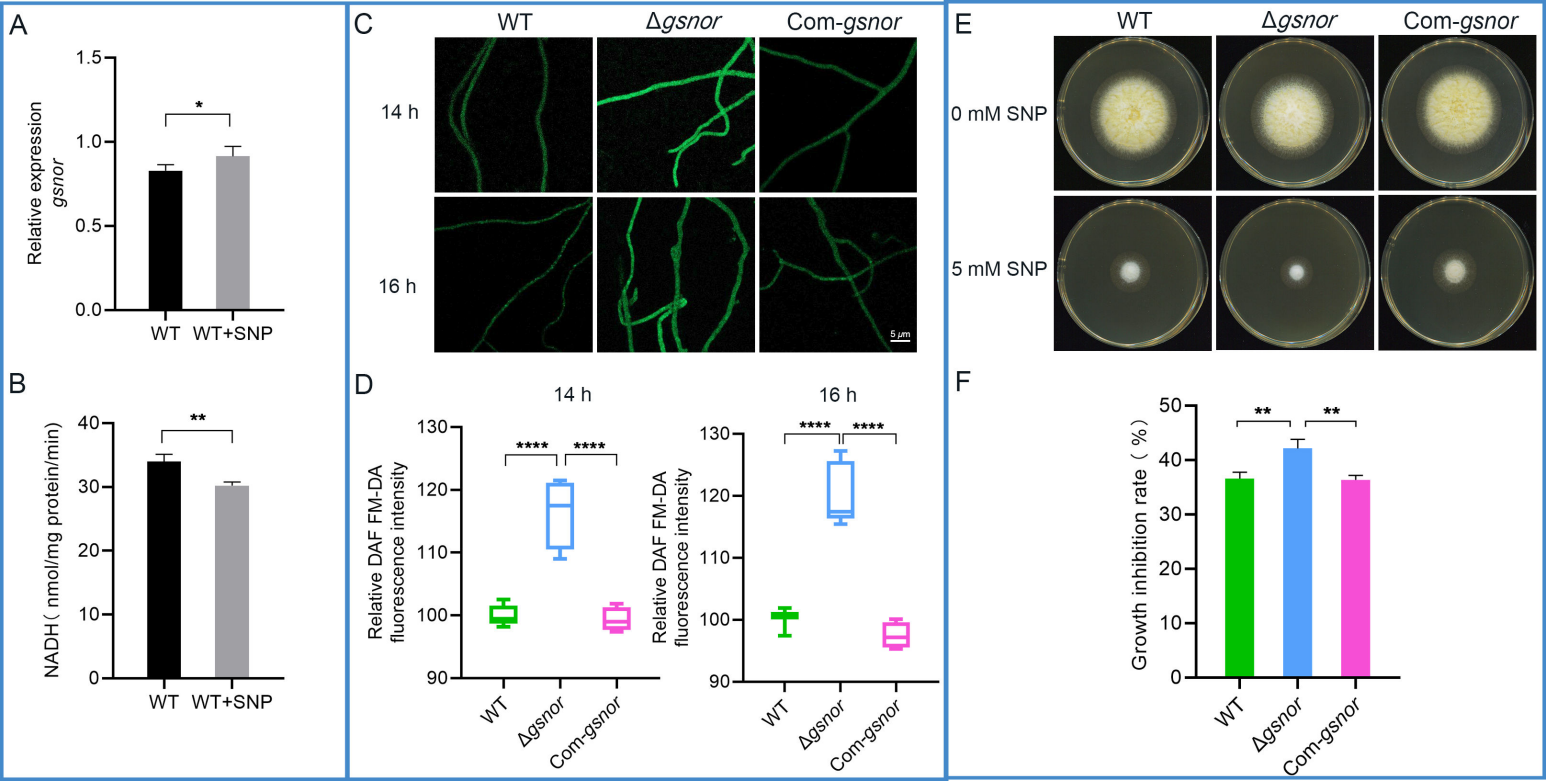
GSNOR is required for NO homeostasis. (**A**) The effect of SNP on *gsnor* expression in the wild type. (**B**) The effect of SNP on GSNOR enzyme activity in the wild type. (**C**) Influence of *gsnor* mutation on the NO level. Vegetative hyphae were stained with the NO-sensitive fluorescent dye DAF FM-DA and observed using a laser scanning confocal microscope. (**D**) Intracellular NO levels were quantified using relative fluorescence intensity. (**E**) Effects of exogenous stresses on the growth of *A. flavus*. A 5 mM SNP was used for exogenous NO stress, with no SNP added as the control. (**F**) Growth inhibition rate under SNP. Growth inhibition rate (%) = (colony diameter under no stress conditions – colony diameter under stress conditions)/colony diameter under no stress conditions × 100. Asterisks indicate significant differences, *P < 0.05, **P < 0.01, ****P < 0.0001.

To explore whether the activity of GSNOR in *A. flavus* is influenced by NO levels, the wild-type strain NRRL 3357 was inoculated into liquid YES medium and cultured for 24 h. Following our previous findings that 500 μM SNP significantly decreases aflatoxin production (24), the same concentration of SNP was added as an exogenous NO donor, and the cultures were incubated for another 24 h. The expression level of *gsnor* and GSNOR enzyme activity in the mycelia was subsequently measured. Quantitative analysis of *gsnor* showed that exogenous SNP treatment significantly upregulated *gsnor* expression (Fig. 3A). However, SNP treatment significantly reduced GSNOR enzyme activity in *A. flavus* (Fig. 3B), which demonstrated that the activity of GSNOR in *A. flavus* was affected by exogenous NO.

To investigate the role of GSNOR in NO homeostasis, the NO levels in *gsnor* deletion mutant, complemented strain, and the wild-type strain were assessed by fluorescent dye DAF-FM DA. The vegetative hyphae from all tested strains were stained with the DAF-FM DA at 14 and 16 h of cultivation on the cover slips, and intracellular NO levels were quantified based on relative fluorescence intensity. The results indicated that at 14 and 16 h, the NO levels in the Δ*gsnor* mutant were significantly elevated, with relative fluorescence intensities increasing by 59.5% and 37.5%, respectively, compared to the wild type (Fig. 3C and D).

To investigate the NO stress response of the GSNOR deletion strain, we assayed the mycelial growth of the strain under SNP. In the presence of SNP, mycelial growth of Δ*gsnor* was significantly reduced, with a higher inhibition rate than wild type and Com-*gsnor*, which suggests that deletion of *gsnor* makes the strain more sensitive to the exogenous NO stress (Fig. 3E and F). These results demonstrate that GSNOR plays an important role in NO homeostasis.

### GSNOR deletion impairs mycelial growth, conidiogenesis, and sclerotial development

**Fig 4:**
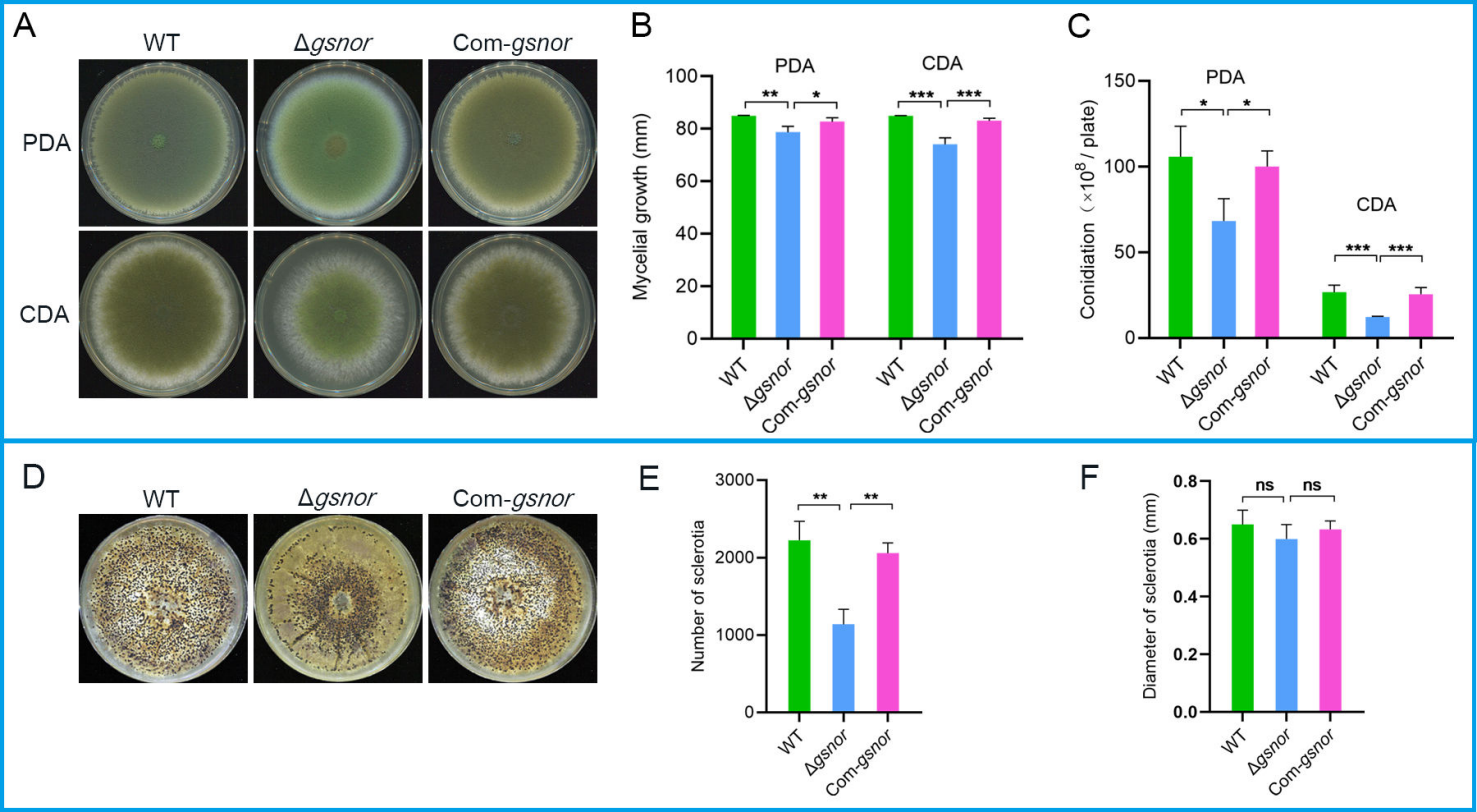
The effect of GSNOR on the growth and sclerotial development of *A. flavus*. (**A**) Phenotype of mycelia grown on PDA and CDA media. All tested strains were inoculated on 90 mm PDA and CDA plates at 30°C for 7 days and then photographed. (**B**) Mycelia growth rate analysis. Colony diameters of the tested strains on PDA and CDA media were measured and analyzed. (**C**) The conidia produced per plate by the tested strains were numbered. A total of 1 × 10^4^ spores were cultured on PDA and CDA media at 30°C for 7 days. (**D**) Sclerotial morphology. A total of 1 × 10² spores were seeded on a WKM plate and incubated at 30°C for 10 days in the darkness. (**E**) Number of sclerotia. (**F**) Diameter of sclerotia. Asterisks indicate significant differences, *P < 0.05, **P < 0.01, ***P < 0.001. “ns” indicates not significant.

To investigate the role of *gsnor* on mycelium growth and conidiogenesis in *A. flavus*, the colonial diameter of the mutant strain Δ*gsnor*, wild-type strain NRRL 3357, and the complemented strain Com-*gsnor* were compared on PDA and CDA media. We found that mycelial growth of Δ*gsnor* was significantly retarded on both PDA and CDA compared to the wild type, with the colony diameter reduced by 7.3% and 9.7%, respectively (Fig. 4A and B). Moreover, the conidia production of Δ*gsnor* was significantly reduced on both media, with a decrease of 35.5% and 53.6%, respectively, compared to the wild type (Fig. 4C). While conidiogenesis of the complemented strain is comparable to that of the wild type. Furthermore, we investigated the impact of GSNOR on sclerotial development in *A. flavus*. Our results indicated that the number of sclerotia significantly decreased in the GSNOR-deficient strain compared to both the wild-type and complemented strains, with reductions of 48.9% and 44.7%, respectively (Fig. 4D and E). However, no significant effect on the sclerotium diameter was observed (Fig. 4F).

### GSNOR deletion impairs conidial germination

**Fig 5:**
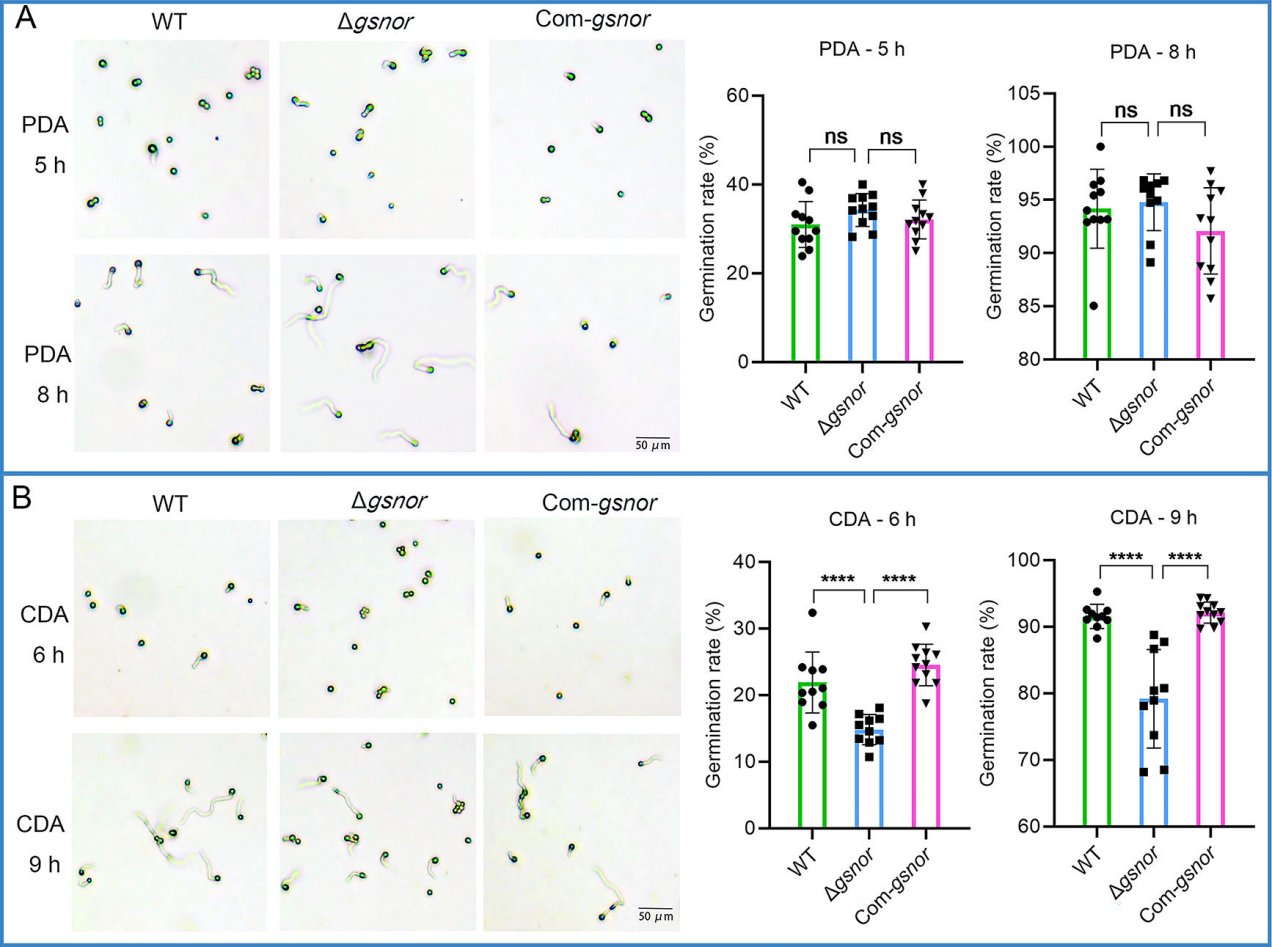
The effect of GSNOR deletion on the conidial germination. (**A**) Germination morphology and germination rate from all indicated strains on PDA media. (**B**) Germination morphology and germination rate from all indicated strains on CDA media. Asterisks indicate significant differences, ****P < 0.0001. “ns” indicates not significant.

To ascertain the role of GSNOR in *A. flavus* conidia germination, the mutant strain, wild-type, and complemented strain were cultured on coverslips covered with a thin layer of PDA or CDA media. Two time points were assessed: one at the initiation of conidia germination and the other when the majority of conidia had germinated. The results showed that on PDA media, no significant difference in conidial germination rate was observed between Δ*gsnor* and the wild type at both 5 and 8 h (Fig. 5A). The conidia germinated more slowly on CDA media than on PDA. There was no significant difference in conidial germination rate between the wild-type and Com-*gsnor* strains at 6 and 9 h. In contrast, Δ*gsnor* showed significantly lower germination rates compared to the wild type, with reductions of 32.3% and 13.5% at 6 and 9 h, respectively (Fig. 5B). These results suggest that GSNOR plays a role in *A. flavus* conidial germination in certain specific conditions.

### GSNOR deletion reduces AFB1 biosynthesis

**Fig 6:**
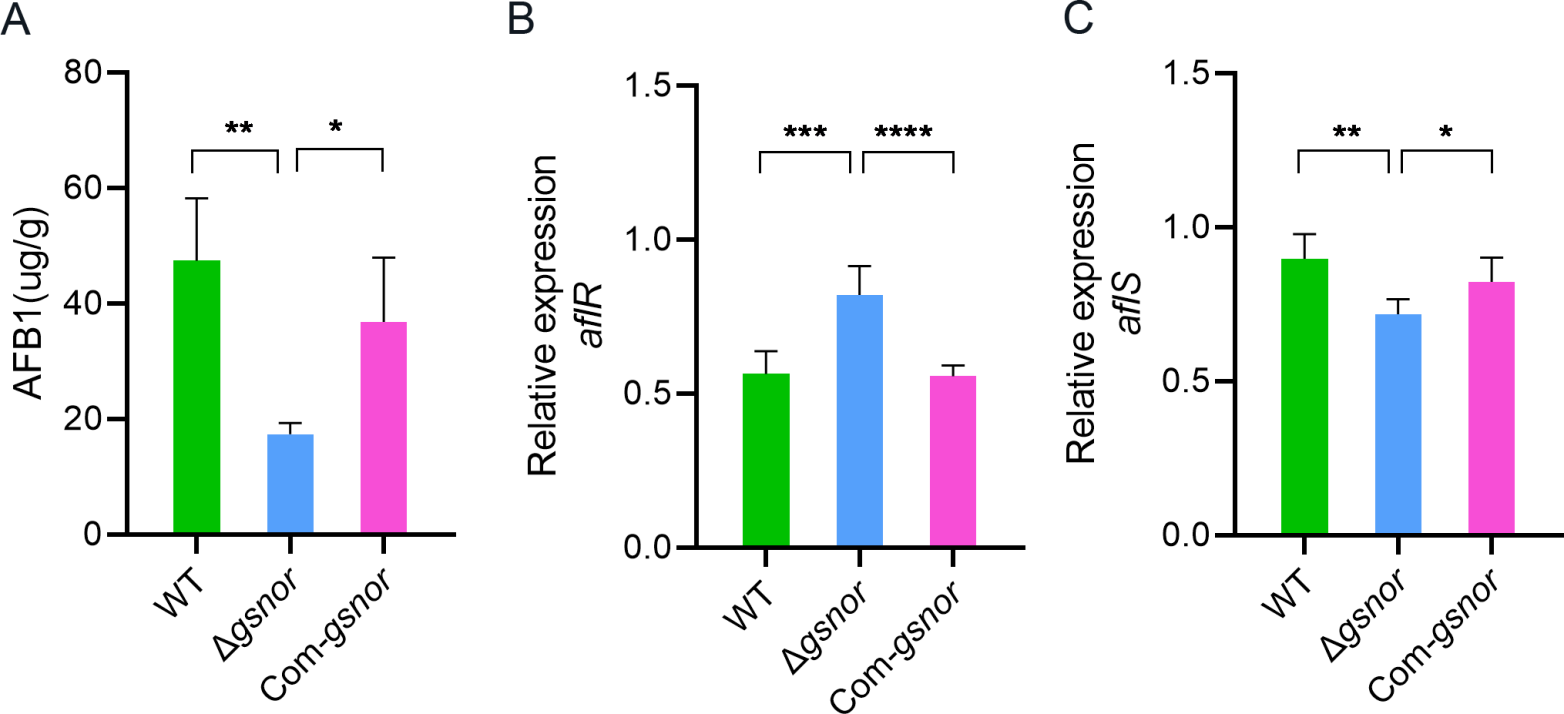
Analysis of the biosynthesis of aflatoxin by *A. flavus* strains. (**A**) Aflatoxin production by the *A. flavus* strains. Conidia of the indicated strains were inoculated on YES media. The production of AFB1 was determined using HPLC after 4 days of incubation at 28°C. The relative levels of expression of *aflR* (**B**) and *aflS* (**C**) in all indicated strains were also quantified. Asterisks indicate significant differences, **P < 0.01, ***P < 0.001, ****P < 0.0001.

AFB1 is a notorious secondary metabolite produced by *A. flavus*. We found that GSNOR deletion resulted in a significant reduction in AFB1 production, which decreased by 63.4% compared to the wild-type strain. In contrast, the complemented strain showed no significant difference in toxin production when compared to the wild type (Fig. 6A). Furthermore, the expression level of the key transcription factor AflR and its regulatory cofactor AflS in the AFB1 biosynthesis pathway was analyzed. Curiously, the expression of *aflR* was significantly elevated in Δ*gsnor* compared to the wild type, while *aflS* expression was reduced by 20% (Fig. 6B and C). The results forecast that the impact of GSNOR on aflatoxin synthesis may not be limited to the transcriptional level, and other regulatory mechanisms may also be involved.

### GSNOR is required for pathogenic development in *A. flavus*

**Fig 7:**
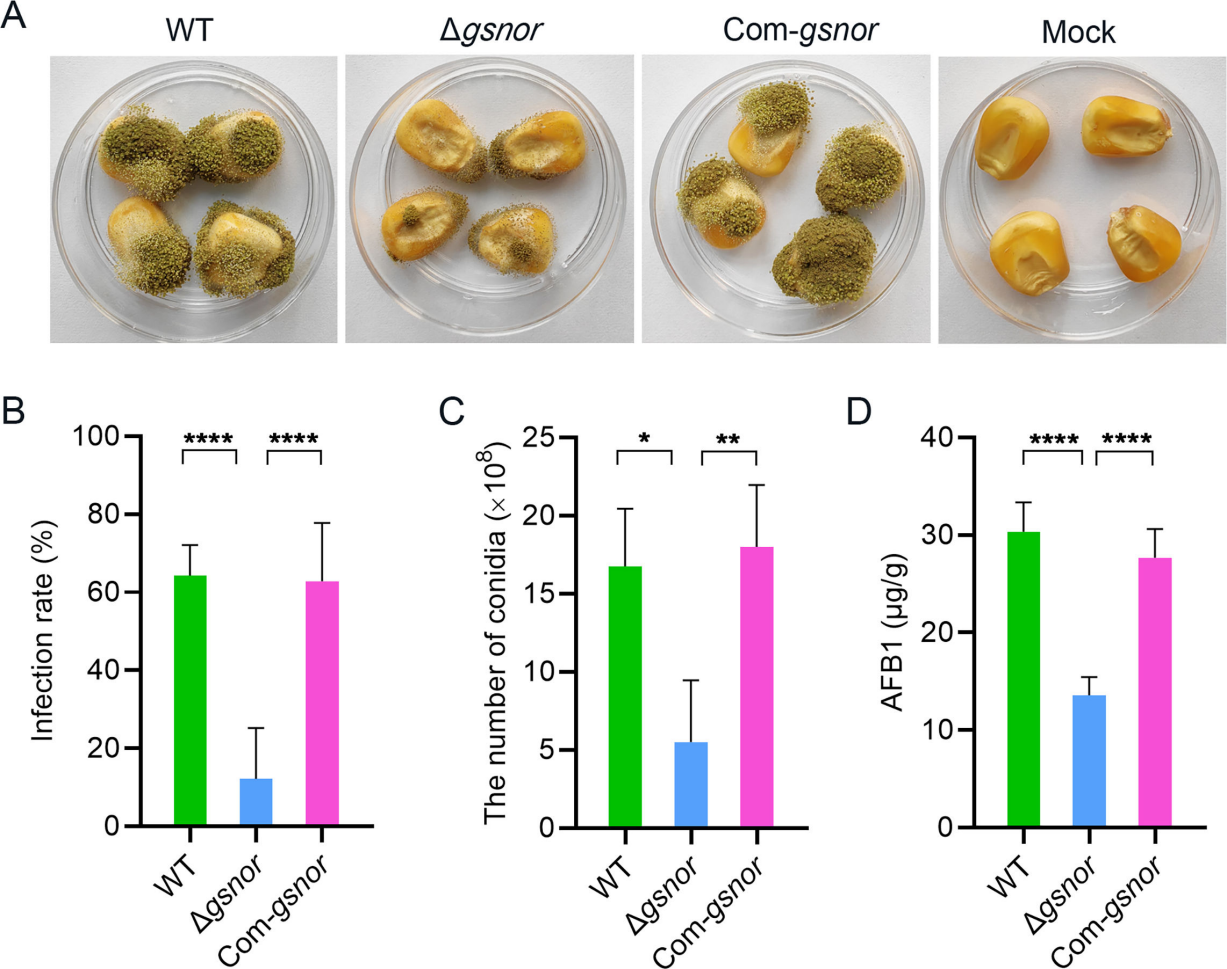
Pathogenicity assays. (**A**) Virulence assay of mutants on maize kernels. (**B**) Infection rate on kernels. (**C**) Conidial production on kernels. (**D**) AFB1 levels in infected kernels. Asterisks indicate significant differences, *P < 0.05, **P < 0.01, ****P < 0.0001.

To understand the role of GSNOR in pathogenesis, conidia suspension of all tested strains was inoculated with maize kernels. Compared with the wild-type and the complemented strains, the virulence of ∆*gsnor* was significantly reduced on kernels. At 10 dpi, in contrast to numerous conidia on the kernel surface caused by the wild-type and Com-*gsnor* strain, Δ*gsnor* produced fewer conidia (Fig. 7A). The infection rate and conidia production of the Δ*gsnor* strain were reduced by 81% and 67.2%, respectively, compared to the wild type (Fig. 7B and C). Additionally, aflatoxin content in the infected kernels was analyzed, and the results showed that the AFB1 levels in the kernels infected by the Δ*gsnor* strain were significantly lower, with a 55.1% reduction compared to those infected by the wild-type strain (Fig. 7D). The Com-*gsnor* strain showed no significant difference compared to wild type in terms of infection rate, conidial production, and AFB1 level. This suggests that GSNOR plays an important role in kernel invasion and colonization.

### GSNOR is essential for ROS balance in *A. flavus*

**Fig 8:**
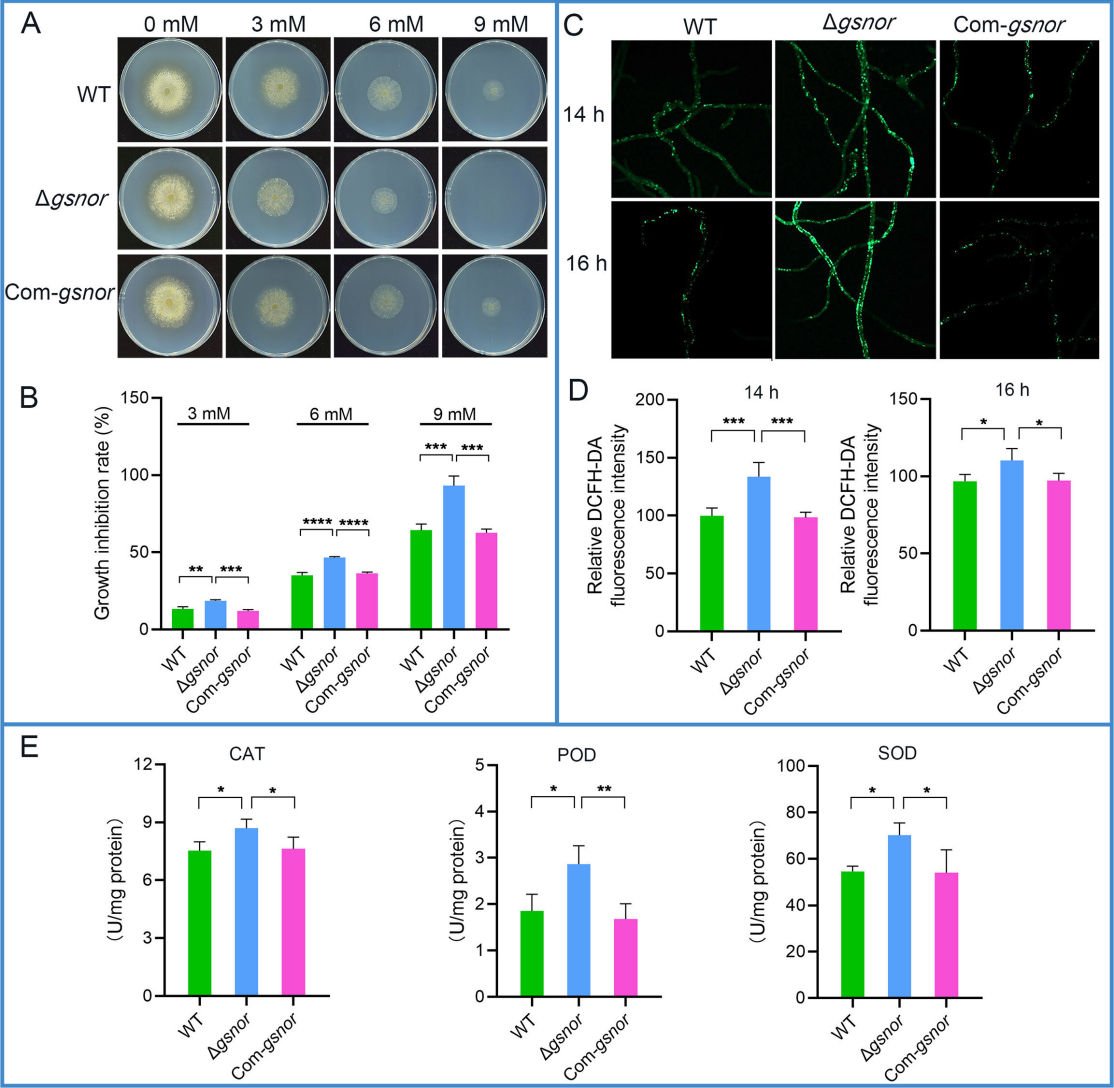
GSNOR is required for ROS balance of *A. flavus*. (**A**) Effects of H_2_O_2_ on the growth of *A. flavus* strains. Colonies of mutants subjected to H_2_O_2_ stress. (**B**) Growth inhibition rate of all indicated strains under 3, 6, and 9 mM H_2_O_2_. (**C**) Analyses of ROS levels in hyphae by DCFH-DA probe. (**D**) Relative fluorescence intensity analysis. (**E**) Antioxidant enzyme activity analyses, including CAT, POD, and SOD. Asterisks indicate significant differences, *P < 0.05, **P < 0.01, ***P < 0.001, ****P < 0.0001.

GSNOR is crucial for maintaining redox homeostasis in animals and plants. To determine whether GSNOR serves a similar role in *A. flavus*, oxidative stress assays were performed with H₂O₂. The results revealed that the Δ*gsnor* mutant exhibited increased sensitivity to H₂O₂ compared to both the wild type and Com-*gsnor*. Specifically, the growth inhibition rates of Δ*gsnor* were 1.4, 1.3, and 1.5 times those of the wild type at 3, 6, and 9 mM H₂O₂, respectively. Notably, at 9 mM, Δ*gsnor* displayed almost no growth, while the wild type and Com-*gsnor* showed substantial growth (Fig. 8A and B).

Given that the GSNOR deletion mutant exhibits hypersensitivity to oxidative stress, the ROS levels in the mycelia were subsequently assessed using DCFH-DA staining. At 14 and 16 h, ROS levels in Δ*gsnor* significantly increased, approximately 1.4 and 1.2 times that of the wild type, respectively. No significant differences were observed between Com-*gsnor* and wild-type strain (Fig. 8C and D). These results suggest that GSNOR plays a crucial role in regulating ROS levels in *A. flavus*.

To further investigate the regulatory role of GSNOR in the intracellular antioxidant system, the activities of CAT, POD, and SOD were analyzed. The results showed that the Δ*gsnor* exhibited a significant 15% increase in CAT activity, a 55% increase in POD activity, and a 30% increase in SOD activity compared to the wild type. No significant differences were observed in the activities of these three enzymes between the complemented strain and the wild-type strain (Fig. 8E). This suggests that in Δ*gsnor*, the imbalance of ROS levels requires the activation of antioxidant enzyme activity to restore balance. This further emphasizes the important role of GSNOR in maintaining redox homeostasis in *A. flavus*, which is likely integral to the adaptive regulation of oxidative stress responses.

## DISCUSSION

GSNOR is a critical enzyme in the NO signaling pathway, primarily catalyzing the reduction of GSNO, which indirectly regulates intracellular NO levels. More importantly, GSNOR plays a key role in signal transduction, stress responses, and metabolic regulation. In plants, GSNOR-mediated NO signaling is essential for normal growth and development and is widely involved in responses to both biotic and abiotic stresses. Its function in these processes is primarily mediated through the regulation of NO homeostasis, interaction with ROS, and modulation of oxidative signaling and stress (12, 37).

In this study, a gene encoding GSNOR was identified in *A. flavus* through homology analysis. GSNOR belongs to a class III alcohol dehydrogenase, a family that also includes other dehydrogenases not dependent on GSNO (13). In all species where GSNOR has been identified so far, typically only one GSNOR homolog has been found (21, 38). Through preliminary homology alignment in *A. flavus*, three homologous proteins were identified. However, a more detailed conserved amino acid alignment, particularly at position Cys11, led to the identification of AFLA_058310 as the gene encoding GSNOR. This was further confirmed by the loss of enzyme activity in the knockout strain. In contrast, AFLA_059790 and AFLA_072340 are likely to encode other dehydrogenases that do not depend on GSNO.

GSNOR plays an important role in NO homeostasis in *A. flavus*, which is vital for cell growth, as excessive NO can be toxic. In previous studies, we found that the flavohemoglobin plays a role in maintaining NO homeostasis in *A. flavus*, with its deletion leading to a significant increase in NO levels. Here, we found that the deletion of GSNOR also resulted in elevated NO levels and increased sensitivity to SNP stress, indicating that GSNOR also plays a significant role in NO homeostasis. Interestingly, we observed that the expression of *gsnor* was upregulated, while GSNOR enzyme activity significantly decreased in wild-type *A. flavus* treated with SNP. We predicted that GSNOR activity might be regulated by *S*-nitrosylation modifications. It has been found that in *A. thaliana*, the transcription level of GSNOR1 is not regulated by stress or developmental signals, but GSNO treatment *in vitro* leads to the *S*-nitrosylation of Cys10, which alters GSNOR conformation and affects its stability (39). *A. flavus* GSNOR contains a similarly conserved Cys11, which has been validated in *A. nidulans* as essential for GSNOR enzyme activity *in vitro* (21). However, whether *A. flavus* GSNOR undergoes *S*-nitrosylation remains to be further investigated.

GSNOR is involved in the regulation of *A. flavus* growth and development. In this study, we found that GSNOR is widely expressed across multiple cellular compartments. And it is expressed at various developmental stages of *A. flavus*, including cnidogenesis, spore germination, and mycelial growth. Deletion of GSNOR resulted in slower growth on both PDA and CDA media, with a significant reduction in conidia production compared to the wild type. Spore germination assays revealed that after 9 h on the nutrient-poor CDA medium, the germination rate of the knockout strain was significantly lower than that of the wild type. Pathogenicity tests on maize kernels further revealed that the knockout strain exhibited reduced infection ability compared to the wild type. These findings suggest that GSNOR may regulate *A. flavus* growth and development, thereby affecting its pathogenicity. In *Magnaporthe oryzae*, deletion of MoSFA1 (GSNOR orthologs) also led to slow colony growth, sparse mycelium, reduced melanin synthesis, and few conidia production, as well as significantly attenuated virulence (36). Further research demonstrated that insufficient turgor pressure and significantly reduced penetration ability led to the marked decrease in pathogenicity (40).

GSNOR is involved in the regulation of aflatoxin biosynthesis in *A. flavus*. In our previous study, we demonstrated that exogenous NO at specific levels influenced AFB1 production in *A. flavus*. And in the *fhbA* deletion mutant, endogenous NO levels significantly increased to 4.2 times that of the wild type, while AFB1 production was markedly reduced by 30% (24). A similar phenomenon was observed in *A. nidulans*, where the *fhbA* deletion mutant exhibited an accumulation of NO, leading to a reduced yield of the mycotoxin sterigmatocystin (9). However, there are fewer reports on the involvement of GSNOR in fungal secondary metabolism. In *G. lucidum*, GSNOR was found to be involved in the ganoderic acid biosynthesis. Silencing GSNOR alleviated the increase in GA production (23). This study found that in the deletion mutant, the activity of the GSNOR enzyme was absent, NO levels were elevated to approximately 1.6 times that of the wild type, and AFB1 production was significantly reduced by 67%. However, in the GSNOR deletion mutant, the expression of *aflR* was significantly upregulated by 46%, while *aflS* expression was downregulated by 20%. Despite these changes, toxin production was significantly reduced. This reduction in toxin levels could be attributed to two potential factors: (i) the decreased transcription of *aflS*, an accessory factor of *aflR*, may contribute to the decline in toxin production; (ii) the GSNOR deletion may alter intracellular *S*-nitrosylation levels, thereby modulating toxin biosynthesis.

GSNOR plays a crucial role in the redox homeostasis in *A. flavus*. The interplay between NO signaling and ROS signaling is both tight and complex, with the two often intertwining and regulating each other during cellular physiological processes. Studies have shown that GSNOR indirectly affects ROS generation and clearance by regulating NO homeostasis. Conversely, ROS/RNS regulate GSNOR activity and stability through redox-based post-translational modifications, establishing a dynamic balance (12, 37). On one hand, GSNOR maintains NO homeostasis by degrading GSNO (a storage form of NO), and NO can react with ROS (such as superoxide anion, O_2_^−^) to form peroxynitrite (ONOO^−^), thereby reducing the toxicity of O_2_^−^ and alleviating certain oxidative damage (41). On the other hand, ROS can oxidize the cysteine residues at the substrate-binding site of GSNOR, leading to the loss of catalytic zinc ions, which reduces GSNOR enzyme activity (12, 42). However, the effect of GSNOR on ROS levels varies among different fungal species. In *G. lucidum*, it has been found that silencing GSNOR leads to GSNO accumulation, significantly reducing ROS levels, which is thought to be due to the increased activity of CAT, further accelerating ROS clearance and significantly lowering ROS signaling intensity (23). In *Colletotrichum gloeosporioides*, the deletion of *CgGSNOR* also led to a significant reduction in ROS levels (43). While in *M. oryzae*, deletion of MoSFA1 triggered ROS burst and diminished antioxidant capacity, along with a marked enhancement in the activities of POD and SOD (36). Similarly, we found that deletion of GSNOR in *A. flavus* resulted in a significant increase in ROS levels, along with a marked enhancement in the activities of CAT, POD, and SOD enzymes. We speculate that the elevated NO levels caused by GSNOR deletion led to an explosion of ROS, which subsequently triggers the activation of the cell’s antioxidant defense mechanisms to maintain redox homeostasis. The oxidative stress experiments confirmed that the imbalance in ROS homeostasis in the GSNOR-deficient strain makes it more sensitive to H₂O₂, suggesting that GSNOR plays a pivotal role in maintaining ROS homeostasis and responding to oxidative stress in *A. flavus*. We further speculate that the decrease in aflatoxin levels in the GSNOR deletion strain is closely associated with increased ROS levels. Because aflatoxin biosynthesis may serve as a novel source of ROS—a potential redox signal to initiate resistance to oxidative stress, and fungal sensitivity to hydrogen peroxide treatment is typically inversely proportional to aflatoxin production (44).

In all, GSNOR plays a crucial role in NO homeostasis and the ROS balance in *A. flavus*. Loss of GSNOR leads to elevated NO and ROS levels, increasing sensitivity to NO and oxidative stress. Additionally, GSNOR deletion impairs conidiogenesis, conidia germination, mycelial growth, sclerotial development, pathogenicity, and notably reduces aflatoxin production. The precise mechanisms by which GSNOR regulates fungal growth, development, and secondary metabolism—either through *S*-nitrosylation or ROS signaling—warrant further investigation. Regardless, this study highlights the potential of targeting GSNOR and other factors in the NO signaling pathway for early prevention of aflatoxin contamination in food.
